# Comment on: ‘Impact of home-based follow-up care on hypertension control among patients attending Thika Level Five Hospital, Kiambu County, Kenya: a randomized controlled study’

**DOI:** 10.1093/inthealth/ihag007

**Published:** 2026-02-10

**Authors:** Benedict Kusi Ampofo, Marian Sapara Arthur, Solomon Gyabaah

**Affiliations:** Directorate of Internal Medicine, Komfo Anokye Teaching Hospital, P.O. Box 1932, Adum, Kumasi, Ghana; Department of Pharmacology, School of Pharmacy and Pharmaceutical Sciences, University of Cape Coast, P.O. Box CCLN 33, Cape Coast, Ghana; Directorate of Internal Medicine, Komfo Anokye Teaching Hospital, P.O. Box 1932, Adum, Kumasi, Ghana

We refer to the randomised controlled trial by Njaramba et al. evaluating a home-based follow-up strategy for blood pressure control among adults with hypertension in Kenya.^1^ The study addresses an important public health problem and contributes to ongoing discussions on how community-supported approaches may strengthen hypertension care in low- and middle-income settings.

We offer a small number of observations that may help place the findings in a clearer context.

It would be helpful for readers to have a clearer insight into the primary hypothesis the trial was designed to test and the assumptions used to determine the sample size, in line with CONSORT (Consolidated Standards of Reporting Trials) reporting guidelines for randomised trials.^2^ In randomised trials, sample size calculations are usually based on the expected treatment effect, how much the outcome varies between participants and the desired statistical power.^3^ For continuous outcomes such as blood pressure, this variation is commonly expressed as the standard deviation, while for binary outcomes (e.g. controlled vs uncontrolled blood pressure), it depends on the expected proportion of participants achieving control.^3^ Stating the anticipated treatment effect and how these assumptions informed the sample size would help to clarify what the study was designed to detect and how much confidence to place in the observed results. This is particularly important when multiple outcomes are assessed and when large treatment effects are reported.

The trial includes repeated measurements of blood pressure within individuals over time, yet the analytical approach relies largely on cross-sectional comparisons and simple statistical tests. Methods that adjust for baseline values and account for within-participant change may provide more robust and precise estimates of treatment effects.^4–7^ Approaches such as analysis of covariance, linear mixed-effects models or generalised estimating equations are commonly used in this setting and allow appropriate handling of correlated observations over time.^4–7^ Clarifying whether analyses followed an intention-to-treat framework, and how missing data were addressed, would help readers to better understand how the analyses were conducted and how robust the findings are.

The intervention represents a complex, multicomponent strategy, combining home visits, text message reminders, counselling and a structured follow-up. While such approaches reflect real-world practice, they make it difficult to determine which elements were the most influential in producing the observed effects. Established frameworks for evaluating complex interventions, such as those developed by the Medical Research Council, emphasise the importance of accompanying outcome evaluation with basic process measures.^8^ In this context, reporting how many counselling sessions participants completed, whether key messages were understood, or whether text messages were received and acted upon, would provide insight into how participants engaged with the intervention. Without such information, it is harder to determine whether improvements were driven by counselling content, the frequency of contact, reminders, or their combined effect. These details are important for interpreting the findings and for considering how the intervention might be replicated or adapted in other settings.

Despite these observations, the study adds to a growing body of evidence supporting community-oriented approaches to hypertension care in sub-Saharan Africa. We recognise the considerable challenges of conducting randomised trials in low-resource settings, including constraints related to staffing, follow-up and implementation complexity. Community-based strategies such as those evaluated in this study are therefore both necessary and promising. Precisely for this reason, clear framing of the study design and analytical approach, together with a clearer description of how the different components of the intervention were delivered and taken up by participants, is important to place the findings in context. Such clarity can help to ensure appropriate interpretation and support future work intended to inform policy decisions and scale-up in similar settings where opportunities for replication may be limited.

## Data Availability

No new data were generated or analysed for this article.
